# Combining Oxymatrine or Matrine with Lamivudine Increased Its Antireplication Effect against the Hepatitis B Virus *In Vitro*

**DOI:** 10.1155/2013/186573

**Published:** 2013-01-21

**Authors:** Zhi-Jie Ma, Qi Li, Jia-Bo Wang, Yan-Ling Zhao, Yan-Wei Zhong, Yun-Feng Bai, Rui-Lin Wang, Jian-Yu Li, Hui-Yin Yang, Ling-Na Zeng, Shi-Biao Pu, Fei-Fei Liu, Da-Ke Xiao, Xin-Hua Xia, Xiao-He Xiao

**Affiliations:** ^1^China Military Institute of Chinese Medicine, 302 Military Hospital, Beijing 100039, China; ^2^College of Traditional Chinese Medicine, Chengdu University of Traditional Chinese Medicine, Chengdu, Sichnan 610075, China; ^3^Pediatric Liver Disease Therapy and Research Center, 302 Military Hospital, Beijing 100039, China; ^4^Integrative Medical Center, 302 Military Hospital, No. 100 4th Ring Road, Beijing 100039, China; ^5^College of Pharmacy, China Pharmaceutical University, Nanjing, Jiangsu 211198, China; ^6^College of Pharmacy, Hunan University of Chinese Medicine, Changsha, Hunan 410208, China

## Abstract

Some recent clinical reports have shown that the combination of oxymatrine, a phyto-derived drug, with lamivudine (3TC) could improve its curative effect against hepatitis B virus (HBV) infection. However, the experimental data in support of this combination strategy are lacking. In this study, we investigated the anti-HBV activity of the combination of 3TC and either oxymatrine or matrine on HepG2 2.2.15 *in vitro*. The activities of the combination and the solo compound, each in different concentrations, were compared on the 3rd, 6th, and 9th experimental days. The cytotoxicity results showed that the nontoxic concentrations of both oxymatrine and matrine to HepG2 2.2.15 cells were 800 **μ**g/mL. We found that the single use of oxymatrine below 100 **μ**g/ml, matrine below 200 **μ**g/ml, and 3TC below 30 **μ**g/ml showed weak inhibitory effects on the secretion of hepatitis B surface antigen (HBsAg), hepatitis B e antigen (HBeAg), and HBV-DNA in culture media; the combination of 3TC (30 **μ**g/ml) with oxymatrine (100 **μ**g/ml) or matrine (100 **μ**g/ml) showed significant inhibitory effects that were higher than or equivalent to the single use of 3TC at 100 **μ**g/ml. The results provide a new impetus to develop novel, multicomponent anti-HBV drugs through the combination of natural products with nucleoside analogs to enhance their activity.

## 1. Introduction

Infection with hepatitis B virus (HBV) is a critical health problem worldwide. Despite the existence of HBV vaccines, approximately 5% of the world population is infected by HBV. China has the largest population of HBV-infected people in the world and is confronting this large disease burden with efficient antiviral drugs. Although there are several new anti-HBV drugs, such as entecavir and tenofovir, which have been recently developed in western countries, there are still challenges to be faced ahead of the widespread adoption of these new drugs in China due to cost constraints. Lamivudine (3TC), formerly one of the widely used nucleos(t)ide analogs (NAs), is no longer recommended as a first line therapy for HBV infection in the guidelines of both the American Association for the Study of Liver Diseases (AASLDs) [1, 2] and the European Association for the Study of the Liver (EASL) [3, 4] due to its defects, such as the emergence of 3TC-resistanant HBV. However, 3TC is still frequently used in China due to its lower price point; this is especially true for the undeveloped regions of China. Obviously, there would be problems with the single use of 3TC; as has been reported in the literature, a single-therapy treatment employing solely 3TC may result in the emergence of both viral drug-resistance and dose-dependent side effects [5].

**Figure 1:**
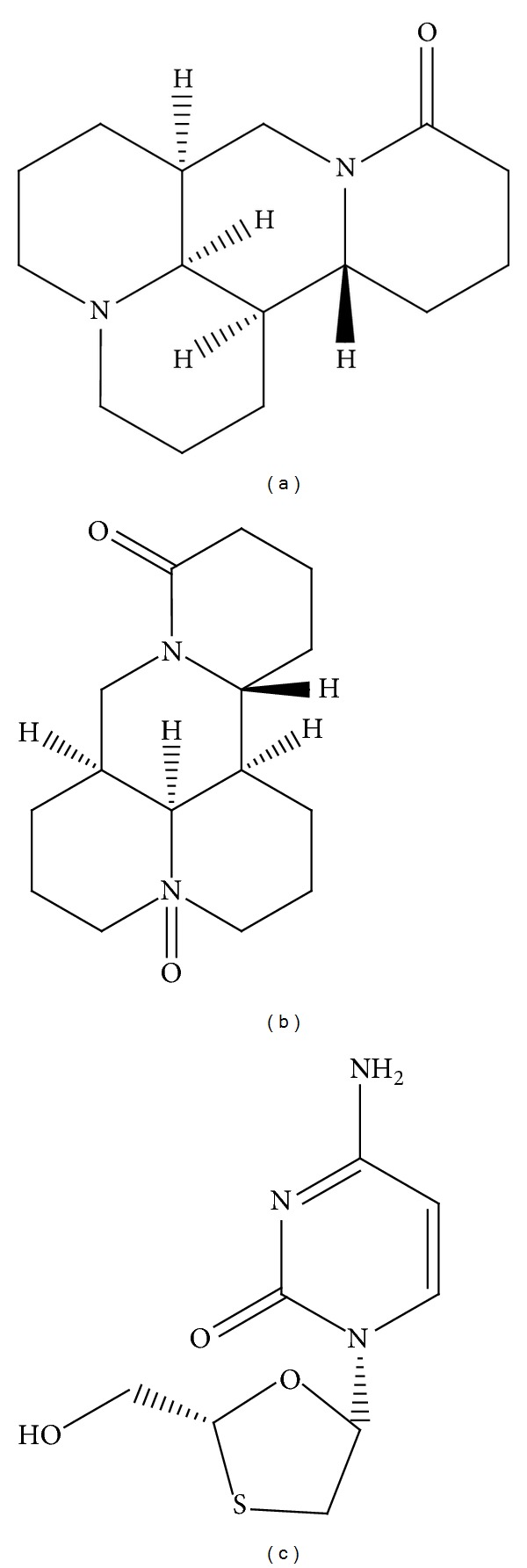
The chemical structures of matrine (a), oxymatrine (b), and lamivudine (c).

**Figure 2:**
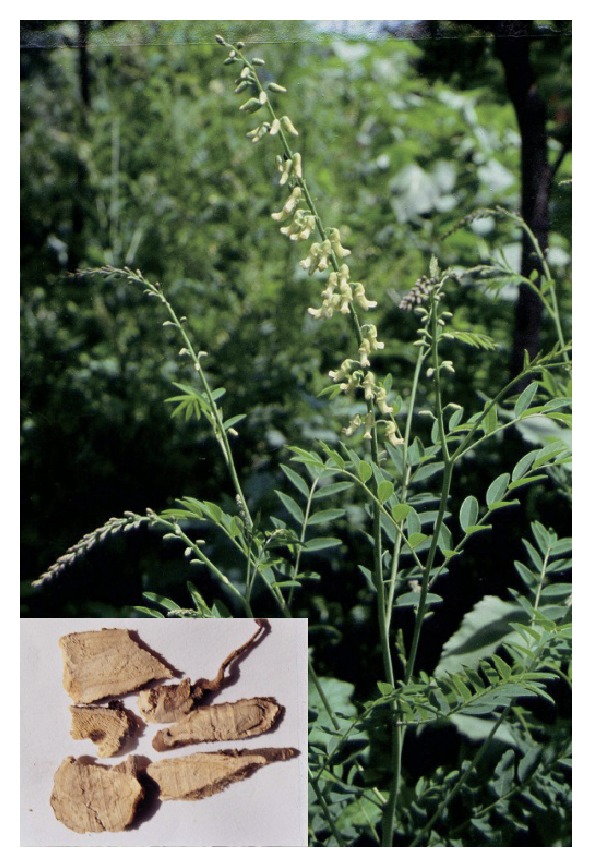
Photographs of *Sophora flavescens* Alt. and the dried root of the plant (left corner).

Under this circumstance, some physicians have attempted to use combinations of traditional Chinese medicines (TCMs) with 3TC to enhance its curative effect. Moreover, the prices of such combinations are relatively cheap compared with the newly launched NAs. We recently noted that the clinical combination of oxymatrine, a phyto-derived drug, with 3TC could improve 3TC's curative effect with regard to HBV infection (Table 1) [6–13]. It has been reported that the combination of oxymatrine with 3TC could significantly increase the negative conversion rate of HBV-DNA and hepatitis B e antigen (HBeAg) in patients compared with the solo use of 3TC. It was also reported that oxymatrine could also decrease the development of drug resistance to 3TC [14–17]. Furthermore, oxymatrine and its analog matrine (Figure 1), the two major alkaloid components in the root of *Sophora flavescens* Alt. (*Kushen* in Chinese, Figure 2), have been reported for their effectiveness in treating hepatocyte injury [18], liver fibrosis [19], and tissue inflammation [20]. Despite the clinical potential of this combination, which has been suggested previously in the literature, there is still a lack of experimental evidence in support of the superiority of the combination of 3TC with oxymatrine or matrine over 3TC alone. Therefore, in this study, we evaluated the anti-HBV effects of the combination of matrine or oxymatrine with 3TC in the human HBV-transfected cell line HepG2 2.2.15 to illustrate the experimental basis for the combination therapy of natural products with NAs at the cellular level.

## 2. Materials and Methods

### 2.1. Drugs and Reagents

Matrine (purity: 99%) and oxymatrine (purity: 99%) were purchased from the National Institutes for Food and Drug Control, Beijing, China. 3TC (purity: 98%) was provided by 302 Military Hospital, Beijing, China, and was used as the positive control. The drugs were dissolved in culture media at certain concentrations before use. 

### 2.2. Cell Culture and Treatment

HepG2 2.2.15 cells, an HBV-transfect human HepG2 cell line, were provided by the Viral Research Lab at the Institute of Contagious Diseases in the 302 Military Hospital. The HepG2 2.2.15 cells were routinely cultured in Dulbecco's modified Eagle's medium (DMEM; Gibco, Langley, OK, USA) supplemented with 10% (v/v) fetal calf serum (Gibco, Langley, OK, USA), antibiotics (100 units/mL penicillin/streptomycin) and 380 *μ*g/mL G418 at 37°C in a humidified incubator with 5% CO_2_.

HepG2 2.2.15 cells were plated at a density of 1 × 10^5^ cells/mL into 24-well plates and incubated for 24 h. Different concentrations of matrine (100, 200, 400, or 800 *μ*g/mL), oxymatrine (100, 200, 400, or 800 *μ*g/mL), and 3TC (30 or 100 *μ*g/mL) were added to the culture media. The cell wells were cultured for 9 days in the presence of the tested drugs, and the supernatants were collected every other day. The cell wells without drugs were set as the control. The concentrations of HBsAg, HBeAg, and HBV-DNA in the supernatant were determined on the 3th, 6th, and 9th experimental day. The intracellular concentrations of HBV-DNA were also determined on the 9th experimental day. 

### 2.3. Cytotoxicity Assay

The *in vitro* drug cytotoxicity was assessed by an MTT (3-(4,5-dimethylthiazol-2yl)-2,5-diphenyltetrazolium bromide) assay. Briefly, HepG2 2.2.15 cells (1 × 10^5^ per well) in the log phase were incubated into 96-well plates. Different concentrations of 3TC, matrine, or oxymatrine were added into cell wells and cultured for 9 days to measure their cytotoxicity. After 9 days, MTT solution (10 *μ*L at 5 mg/mL) was added to each well and incubated for 4 h. Then, the optical density (OD) value was read at 560 nm to measure the amount of cell proliferation using a plate reader (Synergy H1 Hybrid Reader, BioTek, Winooski, VT, USA). Each assay was repeated a minimum of three times. The cell viability was expressed as a percentage of the control. 

### 2.4. Detection of HBsAg and HBeAg

HBsAg and HBeAg in the culture media were determined using ELISA kits (Kehua Biological Technical Co. Ltd., China). The inhibition ratio was determined as follows:

 (1 − absorbance with drug/absorbance without drug) × 100%. Each assay was repeated a minimum of three times.

### 2.5. Detection of HBV-DNA

HBV-DNA in culture supernatants on the 3th, 6th, and 9th days was quantitated by the real-time quantitative PCR analysis. The DNA in the culture supernatants (100 *μ*L, isolated at 5,000 rpm for 5 min in an Eppendorf microcentrifuge) was extracted using the QIAamp DNA Mini Kit (QIAGEN GmbH, Hilden, Germany), following the manufacturer's recommendations. The PCR primers and probe were designed using Primer Express software (Applied Biosystems, Foster City, CA, USA). The primer sequences are shown in Table 2. Amplification was performed in a 50 *μ*L reaction mixture. After the preparation of the reaction mixtures in 96-well plates, the plates were centrifuged at 800 rpm for 1 min in a Beckman GPKR swing rotor centrifuge. Amplification and detection were performed with an ABI Prism 7500 Sequence Detection System. The PCR protocol consisted of the following: (1) a single cycle of 2 min at 50°C, followed by 10 min at 95°C and (2) 45 two-step cycles, with 1 cycle consisting of 15 sec at 95°C and 60 sec at 60°C. 

Data were calculated as the inhibition rate to control by the formula: (%  of  control) = 1 − (ET)/(EC) × 100%, where ET and EC indicated the HBV-DNA expression of the tested drugs and the control without drugs, respectively.

## 3. Results

### 3.1. Cytotoxic Effects of Individual Drugs and Combinations

**Figure 3:**
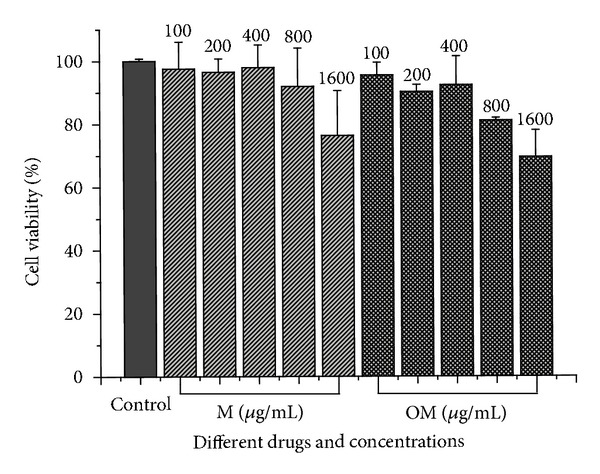
The cytotoxicity of matrine and oxymatrine on HepG2 2.2.15 cells was evaluated by an MTT assay. The cell viability expressed as a percentage of control. The data are presented as the mean ± S.D. (*n* = 3).

The viabilities of the HepG2.2.15 cells in the presence of various concentrations of matrine and oxymatrine were examined with an MTT assay. The results showed that individual drugs at all the detected concentrations had low toxicity to HepG2.2.15 cells (Figure 3). Therefore, the nontoxic concentrations of oxymatrine and matrine to HepG2 2.2.15 cells were both 800 *μ*g/mL. 

### 3.2. Inhibitory Effects of Matrine and Lamivudine on HBV Antigens and DNA

**Figure 4:**
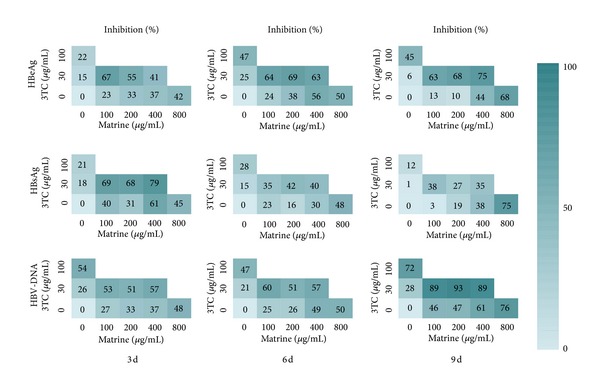
The anti-HBV activity matrices of 3TC, matrine, and their combinations in different concentrations. The average inhibitory effects (%, *n* = 3) on the secretion of HBsAg, HBeAg, and HBV-DNA are labeled in the squares for each pair of combinations. The colors of the squares visually indicate the inhibitory level.

The results of the inhibitory effect of matrine, lamivudine, and their combinations are summarized in Figure 4. After 9 days of treatment, the 100 and 200 *μ*g/mL (0.40 and 0.81 *μ*mol/mL) dose of matrine as a single agent had weak inhibitory effects on the secretion of HBeAg into culture media (13% and 10%, resp.), while the 400 and 800 *μ*g/mL (1.61 and 3.22 *μ*mol/mL) dose had significant inhibitory effects (44% and 68%, resp.). The 30 *μ*g/mL (0.13 *μ*mol/mL) dose of 3TC as a single agent had no significant inhibitory effect on the secretion of HBeAg, while the combinations of 3TC (30 *μ*g/mL) with matrine at 100, 200, or 400 *μ*g/mL all showed significant inhibitory effects (63%, 68%, and 75%, resp.) in a weak, time-dependent manner. Furthermore, the effects of such combinations were all superior to the effects of 3TC in solo use at 100 *μ*g/mL. The results are summarized in Figure 4. 

When matrine was used solo below 400 *μ*g/mL, it showed a weak inhibitory effect on the secretion of HBsAg into culture media after 9 days of treatment. Moreover, the solo use of matrine at 800 *μ*g/mL showed a significant inhibitory effect (75%). It should be noted that the solo use of 3TC at 30 or 100 *μ*g/mL showed weak inhibitory effects on the secretion of HBsAg, while the combinations of 3TC (30 *μ*g/mL) with matrine at 100, 200, or 400 *μ*g/mL showed enhanced, but also weak, inhibitory effects. The inhibitory effects on the secretion of HBsAg into culture media of matrine and 3TC, alone and in combination, decreased gradually with time. 

After 9 days of treatment, the solo use of 400 or 800 *μ*g/mL of matrine showed significant inhibitory effects on the secretion of HBV-DNA into culture media, equivalent to the effect of 3TC in solo use at 100 *μ*g/mL. The solo use of 3TC at 30 *μ*g/mL showed weak inhibitory effects, while the combinations of 3TC (30 *μ*g/mL) with matrine at 100 *μ*g/mL, 200 *μ*g/mL, or 400 *μ*g/mL all showed significant inhibitory effects in time- and dose-independent manners. Furthermore, the effects of such combinations were all greater than the effect of 3TC in solo use at 100 *μ*g/mL.

Either the solo use of matrine and 3TC or their combinations could significantly decrease the intracellular HBV-DNA levels within a certain range of concentrations (Table 3). When matrine was used solo at 400 *μ*g/mL, it showed a weak inhibitory effect on the intracellular HBV-DNA levels. Moreover, the solo use of matrine at 800 *μ*g/mL showed a significant inhibitory effect (94%), equivalent to the effect of 3TC in solo use at 100 *μ*g/mL. The solo use of 3TC at 30 *μ*g/mL also possessed an inhibitory effect of 79%; the combinations of 3TC (30 *μ*g/mL) with matrine at 100 *μ*g/mL, 200 *μ*g/mL, or 400 *μ*g/mL all showed strong inhibitory effects (>94%), which is equivalent to the solo use of 3TC at 100 *μ*g/mL. 

### 3.3. Inhibitory Effects of Oxymatrine and Lamivudine on HBV Antigens and DNA

**Figure 5:**
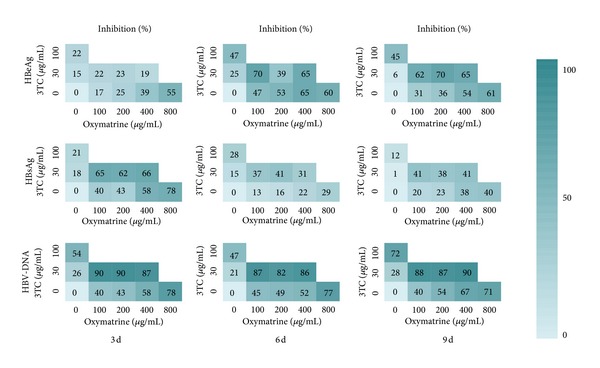
The anti-HBV activity matrices of 3TC, oxymatrine, and their combinations in different concentrations. The average inhibitory effects (%, *n* = 3) on the secretion of HBsAg, HBeAg, and HBV-DNA are labeled in the squares for each pair of combinations. The colors of the squares visually indicate the inhibitory level.

The results of the inhibitory effect of oxymatrine, lamivudine, and their combinations are summarized in Figure 5. After 9 days of treatment, the 100 and 200 *μ*g/mL (0.38 and 0.76 *μ*mol/mL) doses of oxymatrine as a single agent had a weak inhibitory effect on the secretion of HBeAg into culture media (31% and 36%, resp.). The 400 and 800 *μ*g/mL (1.52 and 3.03 *μ*mol/mL) doses of oxymatrine also had evident inhibitive effects on the secretion of HBeAg (54% and 61%, resp.), which were superior to the effect of 100 *μ*g/mL (0.43 *μ*mol/mL) of 3TC (45%). The 30 *μ*g/mL (0.13 *μ*mol/mL) dose of 3TC as a single agent had no significant inhibitory effect on the secretion of HBeAg, while the combinations of 3TC (30 *μ*g/mL) with oxymatrine at 100, 200, or 400 *μ*g/mL all showed significant inhibitory effects (63%, 68%, and 75%, resp.) in a time-dependent and weak dose-dependent manner. Additionally, the effects of such combinations were all better than the effect of 3TC in solo use at 100 *μ*g/mL. 

Oxymatrine had weak inhibitory effects on the secretion of HBsAg into culture as a single agent at all the tested concentrations (100, 200, 400, and 800 *μ*g/mL) after 9 days of treatment. The solo use of 3TC (30 or 100 *μ*g/mL) had a weak inhibitory effect (1% and 12%, resp.); the combinations of 3TC (30 *μ*g/mL) with oxymatrine at 100, 200, or 400 *μ*g/mL also showed evident inhibitory effects (41%, 38%, and 41%, resp.). 

When oxymatrine was used solo at the concentrations of 400 or 800 *μ*g/mL, it showed significant inhibitory effects on the secretion of HBV-DNA into culture media after 9 days of treatment. Such effects are equivalent to the effect of 3TC in solo use at 100 *μ*g/mL. 3TC, as a single agent at 30 *μ*g/mL, also showed a weak inhibitory effect on extracellular HBV-DNA concentrations; the combinations of 3TC (30 *μ*g/mL) with oxymatrine (100, 200, or 400 *μ*g/mL) all showed significant inhibitory effects. Additionally, the effects of such combinations were all superior to the effect of 3TC in solo use at 100 *μ*g/mL. 

The intracellular HBV-DNA inhibition rate of oxymatrine as a single agent at 800 *μ*g/mL was 71%, while its effects below 400 *μ*g/mL were weak (Table 3). The solo use of 3TC at 30 *μ*g/mL possessed an inhibitory effect of 79%. The combinations of 3TC (30 *μ*g/mL) with oxymatrine at 100, 200, or 400 *μ*g/mL all showed strong inhibitory effects (>95%), which is equivalent to the solo use of 3TC at 100 *μ*g/mL.

## 4. Discussion

Traditional Chinese medicine is now widely used to treat hepatitis B in China as well as other areas in the world. Some believe that TCM may offer good therapeutic candidates with special antiviral characteristics to treat HBV infection, while others argue on its scientific merit. Many TCM and related active compounds have been reported to have potent anti-HBV activities, including *Phyllanthus urinaria* L., *Salvia miltiorrhiza* Bge., *Rheum palmatum* L., *Astragalus membranaceus* (Fisch.) Bge., oxymatrine, artemisinin, artesunate, and wogonin [21]. Although there are no effective anti-HBV compounds from TCM being successfully developed into commercial drugs to date, the combination of TCM with NAs represents a potential anti-HBV therapy for future utilization in clinical practice within China. The therapeutic combination of TCM or its components with 3TC is increasingly being reported in the literature [22, 23]. Many researches show that the combination of oxymatrine with 3TC could significantly increase the effect of anti-HBV in chronic hepatitis B treatment. We also found that combinations of oxymatrine together with 3TC had a beneficial clinical response and significantly reduced the drug resistance of 3TC in our hospital (unpublished data). Thus, we have a great interest in the examination of the scientific evidence regarding the therapeutic potential of the combination of matrine or oxymatrine together with 3TC *in vitro*.

The results in this study showed that 3TC at 100 *μ*g/mL (0.44 *μ*mol/mL) had a strong inhibitory effect on either the extracellular or the intracellular levels of HBV-DNA, while its inhibitory effect was weak at 30 *μ*g/mL. 3TC (100 *μ*g/mL) also showed weak inhibitory effects on the extracellular levels of HBeAg and HBsAg. When the concentration of matrine or oxymatrine was below 100 *μ*g/mL, their inhibitory effect was minimal. In contrast, the combination of 3TC (30 *μ*g/mL) with oxymatrine (100 *μ*g/mL) or matrine (100 *μ*g/mL) showed significant inhibitory effects. The inhibitory effects of such combinations on either the extracellular or intracellular levels of HBV-DNA, HBeAg, and HBsAg were higher than the effect of the solo use of each compound at tested concentrations; furthermore, the effects were higher than or equivalent to the effect of the solo use of 3TC at 100 *μ*g/mL (Figures 4 and 5, Table 3). The results illustrate the synergistic potential of the combination of matrine or oxymatrine with 3TC. However, the synergistic effect we discussed in this study is preliminary and superficial, and this result should be further confirmed by the strict evaluation method (e.g., fractional inhibitory concentration index (FICI)) for synergistic effects. The results reported in the aforementioned clinical literatures led us to believe that our results provide experimental evidence at the cellular level that supports the superiority of the combination of 3TC with oxymatrine or matrine. 

**Figure 6:**
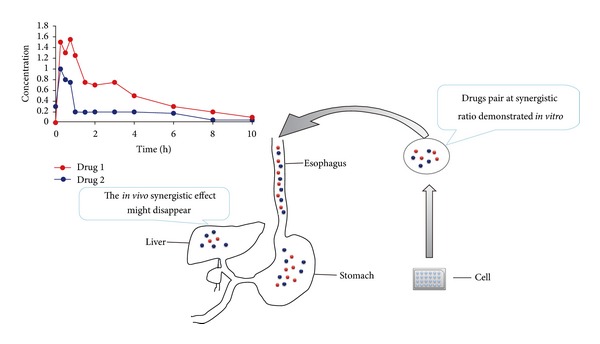
The synergistic effect of the two drugs might be influenced by the metabolic course. When a pair of drug compounds at the desired ratio (demonstrated for synergistic effect *in vitro*) was orally administered into body, the metabolic processes of drug absorption, distribution, metabolism, and excretion will change the ratio of the drugs. As a consequence, the *in vivo* synergistic effects of the drug pair may disappear if the drug pair can only possess a synergistic ratio within a narrow range.

Another phenomenon that piqued our interest is the dose-effect relationships of the combinations. We noted that the combinations of 3TC (30 *μ*g/mL) with matrine or oxymatrine at different concentrations from 100 *μ*g/mL to 400 *μ*g/mL showed a weak, dose-dependent anti-HBV effect. That is, the concentration of 100 *μ*g/mL of matrine or oxymatrine would be enough to produce synergistic effects with 3TC. Furthermore, we can conclude that the synergistic effect between 3TC and matrine or oxymatrine is nearly steady over a relatively wide range of doses, at least from 1 : 1 to 1 : 4. This feature is very important for the clinical application of combinatorial therapy. Different drugs have differential pharmacokinetic characteristics that are time dependent and tissue specific. Although we can combine two synergistic drugs at the correct proportion in one capsule, the two drugs will not achieve the expected proportion in the blood, tissue, cell, or target. Their proportion will certainly vary over time (Figure 6). If the range of the synergistic proportion is narrow, it could be anticipated that the combination will not achieve expected *in vivo* synergistic effects, a phenomenon which has been confirmed *in vitro*. A study of antitumor combination drugs, for example, confirmed this assumption [24]. This study reports two drugs that possess a synergistic effect within a narrow proportion *in vitro*, while the combination had weak effects *in vivo*. When the proportion of the two drugs was purposely controlled in the tumor tissue through a pharmaceutical approach, the combination regained the synergistic effect *in vivo*, similar to the results observed *in vitro*. Therefore, a wide range of synergistic proportions is more advantageous than a narrow range for combinatorial therapy due to pharmacokinetic concerns. It may also be a valuable feature in that there is a relatively wide range of synergetic proportions of 3TC with matrine or oxymatrine for clinical combinatorial therapy. However, the pharmacokinetic differences between 3TC and matrine or oxymatrine have not been fully addressed and require further investigation. 

## Figures and Tables

**Table 1:** Clinical data of the combinational use of oxymatrine with 3TC for the treatment of HBV infection.

Data sources	Groups and dosages	Number of cases	Course of treatment (months)	Rate of ALT normalization (%)	Rate of HBeAg negative conversion (%)	Rate of HBV-DNA negative conversion (%)
Song [6]	3TC(100^#^) + Omt(400)	32	3	78.1	46.9	90.6
	3TC(100)	30	3	50.0	16.7	86.7

Fan [7]	3TC(100) + Omt(400)	32	3	25	15	29
	3TC(100)	30	3	15	5	26

Duan [8]	3TC(100) + Omt(600)	60	6	—	53.3	63.3
	3TC(100)	60	6	—	18.3	23.3

Zhang [9]	3TC(100) + Omt(600)	62	6	—	53.23	62.90
	3TC(100)	62	6	—	19.35	24.19

Xie [10]	3TC(100) + Omt(150)	78	6	100	53.8	55.1
	3TC(100)	42	6	90.5	45.2	47.6

Liu [11]	3TC(100) + Omt(400)	31	12	100	61.3	87.1
	3TC(100)	34	12	100	37.1	85.7

Su [12]	3TC(100) + Omt(300^★^)	30	12	91.23	66.67	86.67
	3TC(100)	30	12	68.26	43.33	63.33

Chen [13]	3TC(100) + Omt(200)	38	12	—	11	23
	3TC(100)	34	12	—	7	16

^#^The dosage unit is mg/day.

^★^The dosage of the former three months is 600 mg/day by intravenous administration, and the dosage of the followed nine months is 300 mg/day by oral administration.

—: the data were not reported in the original literatures.

**Table 2:** The primers used for RT-PCR.

Primer	Sequence	Product (bp)
	F: ACT CGT GGT GGA CTT CTC TCA ATT	
HBV	R: CGC AGA CAC ATC CAG CGA TA	136
	Probe: FAM-AGTCCCCAACCTCCAATCACTCACCA-TAMRA	

	F: GGA AAT CGT GCG TGA CAT TAA G	
*β*-Actin	R: GCT CAT TGC CAA TGG TGA TG	143
	Probe: FAM-TACGTCGCCCTGGACTTCGAGCA-TAMRA	

**Table 3:** The inhibition rates of lamivudine combined with matrine or oxymatrine to the intracellular concentrations of HBV-DNA on the 9th experimental day.

Drugs (*μ*g/mL)	Inhibition (%)	Drugs (*μ*g/mL)	Inhibition (%)
3TC(30)	79		
3TC(100)	97		
Matrine (100)	—	Oxymatrine (100)	—
Matrine (200)	39	Oxymatrine (200)	—
Matrine (400)	59	Oxymatrine (400)	20
Matrine (800)	94	Oxymatrine (800)	71
Matrine + 3TC (100 + 30)	94	Oxymatrine + 3TC (100 + 30)	98
Matrine + 3TC (200 + 30)	95	Oxymatrine + 3TC (200 + 30)	95
Matrine + 3TC (400 + 30)	95	Oxymatrine + 3TC (400 + 30)	98

—: the values are below zero.
